# Structure–Property Relationships in Polyester-Based Polyurethane Foams with Varying Isocyanate Index for Footwear Midsole Applications

**DOI:** 10.3390/polym18151896

**Published:** 2026-08-01

**Authors:** Onder Albayrak, Mehmet Ipekoglu, Omer Uctu, Gonul S. Batibay, Ahmet Calik, Ana Pilipović

**Affiliations:** 1 Department of Mechanical Engineering, Faculty of Engineering, Mersin University, 33343 Mersin, Türkiye; 2 Department of Mechanical Engineering, Faculty of Engineering, Turkish-German University, 34820 Istanbul, Türkiye; ipekoglu@tau.edu.tr; 3 Department of Textile, Clothing, Footwear and Leather, Naci Topcuoglu Vocational School, Gaziantep University, 27600 Gaziantep, Türkiye; omeructu@outlook.com; 4 Research Laboratories Application and Research Center (ALUAM), Turkish-German University, 34820 Istanbul, Türkiye; gonul.batibay@tau.edu.tr; 5 Department of Mechanical Engineering, Faculty of Engineering and Architecture, Burdur Mehmet Akif Ersoy University, 15200 Burdur, Türkiye; acalik@mehmetakif.edu.tr; 6 Department of Technology, Faculty of Mechanical Engineering and Naval Architecture, University of Zagreb, 10002 Zagreb, Croatia; ana.pilipovic@fsb.unizg.hr

**Keywords:** polyurethane foam, footwear midsole, isocyanate index, thermal degradation, mechanical properties, tribological behavior, wear resistance

## Abstract

Polyurethane (PU) foams are widely used in footwear midsoles because their cellular structure, density, impact-attenuation capability, and mechanical durability can be tailored through formulation design. In this study, polyester-based PU foams were prepared at different isocyanate indices while keeping the main formulation components constant, and their structure-property relationships were evaluated under midsole-relevant conditions. The samples were characterized by density, tensile and compression testing, standard abrasion wear testing, water absorption, temperature-dependent flexural resistance, Fourier transform infrared (FTIR), scanning electron microscope (SEM), differential scanning calorimetry (DSC), thermogravimetric analysis/derivative thermogravimetry (TGA/DTG), and dry/wet tribometry. FTIR results confirmed the formation of urethane/urea-related linkages and the absence of detectable residual isocyanate groups, whereas DSC indicated broad heat-flow events typical of segmented PU systems, including high-temperature events that should be interpreted together with TGA. TGA/DTG analysis showed similar initial degradation behavior for all formulations; however, the 138-index sample exhibited the highest *t*_90%_ value, indicating improved high-temperature mass retention. Tribometric tests revealed an environment-dependent coefficient of friction (COF) response: the 138-index sample exhibited the lowest steady-state COF under dry sliding (μ_ss_ = 0.211), whereas the 113-index sample showed the lowest COF value under wet sliding conditions (μ_ss_ = 0.176). Overall, among the three stable formulations investigated, the 113-index formulation exhibited the most balanced multi-property performance. These results suggest that, within the tested formulation range, midsole-relevant PU foam performance is associated with a balance of formulation characteristics rather than simply with increasing the isocyanate index.

## 1. Introduction

Polyurethane is a synthetic polymer formed mainly through reactions between polyols and isocyanates. Since their development, polyurethanes have been used in many fields, including automotive components, packaging, construction, apparel, coatings and adhesives. In segmented polyurethane systems, the stoichiometric balance between isocyanate and polyol components directly affects the hard/soft segment ratio, crosslink density, phase organization, and consequently the mechanical, thermal, and surface-related performance of the final material [1].

The footwear industry, which is a part of the clothing sector, has become an independent sector closely related to fashion today. Shoes, which were considered a basic need in the past, are now shaped by the consumer’s desire for comfort, following fashion trends, and expressing themselves. The supply and demand in the footwear sector are increasing in terms of both quantity and variety worldwide, and the industry continues to grow day by day. According to the “World Footwear Report” prepared by the Portuguese Footwear and Leather Goods Association (APICCAPS) regarding global production and consumption quantities in the sector, it is stated that global footwear production exceeded 22 billion pairs in 2021 [2]. Looking at the world shoe export data, it is seen that China ranked first with approximately 7.9 billion pairs of shoe exports in 2021, followed by Vietnam, Indonesia, and Türkiye. According to the APICCAPS report, the average number of pairs of shoes per person per year worldwide is stated to be four [2].

A shoe consists of several functional components, including the upper, midsole, outsole, and auxiliary accessories. The upper encloses and supports the foot, while the outsole is the outermost layer that provides traction and abrasion resistance against the ground. Positioned between these two components, the midsole serves as the primary cushioning and energy-absorbing layer, playing a critical role in comfort, impact attenuation, and load distribution during walking and running. Due to these functional demands, midsole materials are required to exhibit a balanced combination of low density, elasticity, resilience, and mechanical durability. In addition to these requirements, resistance to wear, moisture exposure, repeated deformation, and frictional interactions under dry and wet contact conditions are also important for performance [3,4].

Midsole materials can be broadly classified as natural or synthetic. While natural materials such as leather and rubber are still used to a limited extent due to their high cost, modern footwear manufacturing predominantly relies on synthetic materials, including ethylene-vinyl acetate (EVA), thermoplastic polyurethane (TPU), expanded thermoplastic polyurethane (E-TPU), polyurethane (PU), and thermoplastic elastomers (TPR) [5]. Among these, polyurethane has gained particular importance as a midsole material due to its highly tunable formulation, favorable mechanical and cushioning properties, low density, and compatibility with industrial-scale molding processes [6,7].

Polyurethane midsoles are typically produced through the controlled reaction of polyol, isocyanate, and catalyst systems, resulting in an exothermic polymerization process [8,9]. The reactive mixture is injected into heated metal molds, where it expands and solidifies into a foam structure. Owing to their cellular morphology, PU-based midsoles offer significant weight reduction compared to dense elastomeric materials while maintaining adequate mechanical strength and energy absorption capacity. Industrial production commonly employs horizontal or rotary polyurethane injection machines. Prior to processing, polyol and isocyanate components are preheated to reduce viscosity and ensure homogeneous mixing. The materials are subsequently combined at high rotational speeds and transferred into aluminum molds, which are maintained at a controlled temperature. After a defined mold dwell time, the molded midsole component is demolded. Silicone-based sprays are generally used to facilitate the removal of products from molds [10], and *N,N′*-dimethylformamide (DMF) is usually used for cleaning molds [11].

In polyurethane midsole manufacturing, key process parameters include raw material preheating temperature, mixing ratio, mixing time, mold temperature, and mold dwell time, as well as the formulation chemistry and mold design [12,13,14,15,16,17]. Studies are being conducted to investigate the effects of production parameters on the mechanical and physical properties of the resulting PU-based midsoles, and efforts are ongoing to develop midsoles suitable for their intended use and purpose. Among formulation-related parameters, the isocyanate index is particularly important because it determines the relative amount of isocyanate available for urethane/urea formation and controls the hard segment content and network rigidity of the polyurethane structure.

Oppon et al. [14] examined the effect of the mixing ratio and preheating temperature of polyol and diisocyanates on the mechanical properties of PU foam. It was observed that as the diisocyanate concentration increased, the foam density, tensile strength, and compressive strength increased. Also, it was stated that foaming time decreased as the preheating temperature increased. In samples obtained with a 50:50 mixing ratio and a preheating temperature of 20–100 °C, it was observed that the use of a preheating temperature of 20 °C increased the tensile and compressive strengths, and there was no significant difference in the tensile and compressive strengths of samples obtained by preheating at 30–100 °C [14]. In a study conducted by Han et al. [18], it was stated that a higher initial temperature of the mold leads to a homogeneous density distribution, while a larger amount of initial injection provides an advantage in obtaining high-density PU foam.

Mohan et al. [19] examined the effects of process temperature on some physical and mechanical properties of PU foam. The temperature range studied was between 25–85 °C, and it was noted that as the temperature increased, the density, axial compressive modulus, and strength decreased. Jackovich et al. [20] investigated the effects of process temperature and mold size on the physical and mechanical properties of PU foam. It was reported that an increase in process temperature and mold size resulted in a decrease in foam density, as well as a decrease in elastic modulus and compressive strength. The impact of mold temperature on the mechanical properties of thermoplastic PU sheets during injection molding was studied by Xiang et al. [21] and it was reported that increasing mold temperature resulted in a decrease in the yield strength and yield strain of the sample.

The study by Linul et al. [22] investigated the effect of density and anisotropy on the quasi-static and dynamic fracture behavior of samples obtained by cutting rigid PU foam panels made from three different density materials. Microstructural analysis of the rigid PU foam panels revealed that high-density PU foams contained smaller and more uniform pores. It was determined that increasing the density of the material used in the rigid PU foam panels significantly increased the mode I fracture toughness. In a study by Horak et al. [23], the effects of ambient temperature, density, and strain rate on the mechanical strength of rigid PU foam were examined. It was noted that in all samples, an increase in ambient temperature resulted in a decrease in tensile modulus, tensile strength, compressive modulus, and compressive strength values, and a similar effect was observed with a decrease in density.

Although the effect of isocyanate index on the mechanical properties of PU foams has been widely studied, the available literature mainly focuses on model formulations, rigid foams, or isolated properties measured under idealized laboratory conditions. In practical midsole applications, PU foams must satisfy multiple and often competing requirements, such as low density, tensile integrity, compression response, abrasion resistance, water exposure tolerance, and flexural durability at different temperatures. Therefore, evaluating how formulation changes associated with different PB/IM ratios influence multiple midsole-relevant properties is important for identifying balanced performance among the formulations tested rather than maximizing a single property. However, the combined mechanical, thermal, wear, and dry/wet coefficient-of-friction (COF) responses of midsole-scale polyester-based PU foam formulations prepared at different PB/IM ratios and corresponding isocyanate indices have not been sufficiently evaluated. In this study, density, tensile strength, compression response, wear resistance, water absorption, and temperature-dependent flexural resistance were evaluated together. In addition, FTIR, SEM, DSC, TGA/DTG, and dry/wet tribometry were used to establish a structure–property framework for evaluating midsole-scale PU foams in terms of chemical structure, thermal stability, surface friction behavior, and cushioning-related performance.

## 2. Materials and Methods

### 2.1. Mold Design

To produce PU midsoles, a PVC shoe last in size 42 was 3D scanned using a scanning device (HDI 100, LMI Technologies, Burnaby, BC, Canada). The resulting scan was converted to .stl format using the FlexScan3D 3.3 software (LMI Technologies, Burnaby, BC, Canada) and transferred to a computer. The last was edited using another software (Autodesk, Delcam Crispin Lastmaker 2016) and converted to an .igs surface format. The midsole design was created using a design program (Rhino 7) according to the last. An aluminum metal mold was designed and produced with a precision manufacturing method that is suitable for the midsole with 7.5 mm thickness. A cooling system was integrated into the mold and a PU midsole production system was established in a laboratory environment.

### 2.2. Sample Preparation

Polyester-based polyurethane (PU) samples were prepared using a two-component system consisting of a polyol blend (PB) and an isocyanate mixture (IM). The polyol blend was formulated by pre-mixing a polyester polyol (KIMPOL^®^ PE 001, Kimpur, Istanbul, Türkiye), 1,4-butanediol (BDO, BASF SE, Ludwigshafen, Germany) as a chain extender, distilled water as a chemical blowing/urea-forming agent, and a tertiary amine catalyst (DABCO^®^ 25 S, Evonik, Essen, Germany). The isocyanate component was composed of monomeric methylene diphenyl diisocyanate (MDI, Kimpur, Istanbul, Türkiye) blended with a linear prepolymer.

The hydroxyl value of the polyester polyol was 56.07 mg KOH/g (functionality f = 2), corresponding to an OH equivalent weight of approximately 1000 g/eq. The BDO exhibited a hydroxyl value of 1240 mg KOH/g, corresponding to an OH equivalent weight of 45 g/eq. Water was considered as a difunctional reactive species with an OH equivalent weight of 9 g/eq. The NCO equivalent weight of the isocyanate mixture used in the stoichiometric calculations was approximately 389 g/eq.

All formulations were prepared with a total batch mass of 40 g. The polyol blend contained 4.0 wt.% BDO, 0.33 wt.% distilled water, and 0.060 g of DABCO^®^ 25 S catalyst per formulation. Since DABCO^®^ 25 S contains hydroxyl-functional 1,4-butanediol as a carrier (OH number = 934 mg KOH/g), its hydroxyl contribution was included in the stoichiometric calculations.

Although polyol blend–to–isocyanate mixture (PB/IM) weight ratios of 45/55, 50/50, 51/49, 52/48, and 55/45 were initially selected for formulation control, the chemical composition of the PU systems was primarily defined and discussed in terms of the isocyanate index.

Stoichiometric calculations were performed by considering the total hydroxyl equivalents contributed by the polyester polyol, BDO, distilled water, and DABCO^®^ 25 S. The isocyanate index (ISO index) was defined as the molar ratio of total NCO equivalents to total OH equivalents multiplied by 100, as given in Equation (1).

ISO index = (NCOeq/OHeq) × 100(1)

The calculated stoichiometric formulation parameters and corresponding recalculated isocyanate index values for all investigated formulations are summarized in Table 1. Total OH-equivalent values and isocyanate indices were recalculated by including the hydroxyl contribution of DABCO^®^ 25 S to ensure consistency with Equation (1).

The production process consisted of sequential Component A preparation, Component B degassing, preheating, mixing, and molding steps. According to the formulation amounts given in Table 1, polyester polyol, 1,4-butanediol (BDO), distilled water, and tertiary amine catalyst (DABCO^®^ 25 S) were weighed using an analytical balance with ±0.001 g precision (Shimadzu ATX224, Kyoto, Japan). Before use, all raw materials were conditioned for 24 h at 23 ± 2 °C in a laboratory conditioning cabinet (TK120, Nuve, Ankara, Türkiye).

For the preparation of Component A, the polyester polyol was transferred into a dry 500 mL glass beaker, and mixing was started at 500 rpm. BDO was then slowly added and mixed at 1500 rpm for 3 min. Distilled water was subsequently added gradually. After water addition was completed, mixing was continued for an additional 3 min. Finally, the catalyst was added. The mixing speed was gradually increased to 2200 rpm, and the mixture was mechanically stirred at 23 ± 2 °C for 10 min to obtain a homogeneous polyol blend, referred to as Component A.

The isocyanate component, referred to as Component B, was transferred to 50 mL centrifuge tubes and subjected to centrifugation at 2000 rpm for 60 s (NF048, Nuve, Ankara, Türkiye) to remove entrapped air bubbles. Subsequently, both Component A and Component B were preheated at 45 °C in an oven (FN120, Nuve, Ankara, Türkiye) for 1 h. Following preheating, Component A was homogenized for 60 s using a mechanical mixer. The required amounts of Component A and Component B were then combined and mixed for 8 s using a mechanical mixer. The reactive mixture was poured into an aluminum mold preheated to 55 °C. A uniform pressing force was applied using a bolt-and-nut system, and the material was kept in the mold for 240 s to complete the reaction. Finally, the mold was opened, and the sample produced was removed.

### 2.3. Characterization

Produced midsole samples containing different “polyol + catalyst” and “isocyanate” mixture ratios were removed from the mold and conditioned for 24 h at 23 °C and 50% relative humidity (TK120, Nuve, Ankara, Türkiye) according to TS EN 12222 [24] prior to characterization. After conditioning, density, tensile strength, compressive response, water absorption, and temperature-dependent flexural resistance were determined. Unless otherwise specified, quantitative measurements were performed on three specimens and reported as mean ± standard deviation. Because of the limited number of replicates, differences among formulations were interpreted as trends unless supported by clearly separated mean values and standard deviations.

#### 2.3.1. Fourier Transform Infrared (FTIR) Spectroscopy

Fourier transform infrared (FTIR) spectroscopy was employed to investigate the chemical structure of the polyurethane samples and to assess the influence of isocyanate index on characteristic functional groups. FTIR analyses were performed using an FTIR spectrometer in the range of 4000–400 cm^−1^ with a resolution of 4 cm^−1^. The spectra were recorded to identify the formation of urethane-related bonds and to examine changes in hydrogen bonding and segmental organization as a function of isocyanate index.

#### 2.3.2. Differential Scanning Calorimetry (DSC) and Thermogravimetric Analysis (TGA)

Differential scanning calorimetry (DSC) was performed to evaluate the heat-flow response of the polyurethane samples as a function of isocyanate index. DSC measurements were carried out under a nitrogen atmosphere up to 450 °C at a heating rate of 10 °C/min, and the reported thermograms correspond to the heating scan. Because polyurethane degradation may begin within the high-temperature DSC range, heat-flow events above the onset of thermal decomposition were interpreted qualitatively and discussed together with the corresponding TGA/DTG profiles rather than assigned solely to reversible segmental transitions.

Thermogravimetric analysis (TGA) was conducted to determine the weight loss behavior and thermal degradation characteristics of the polyurethane samples. TGA measurements were performed under a nitrogen atmosphere up to 800 °C at a heating rate of 10 °C/min. The residual mass and derivative thermogravimetry (DTG) profiles were recorded as a function of the temperature.

#### 2.3.3. Density

The densities of the three samples were obtained by dividing their masses by their volumes. The masses of midsoles prepared using different “polyol + catalyst” and “isocyanate” ratios were measured using an analytical balance (Shimadzu ATX224, Kyoto, Japan). The volumes were determined based on the Archimedes principle using the water displacement method, in which the displaced water volume was recorded and used for volume calculation. All measurements were conducted using distilled water at room temperature (≈23 °C).

#### 2.3.4. Scanning Electron Microscope (SEM) Analysis

For microstructure examination, samples were taken from the same region of the produced midsoles, and after coating the samples with gold, their transverse surfaces were imaged with SEM (Zeiss Gemini SEM 300, Carl Zeiss Microscopy GmbH, Oberkochen, Germany).

#### 2.3.5. Water Absorption Test

Water absorption test was performed on three samples in accordance with TS 5501 [25] standard. Specimens with dimensions of 5 mm × 5 mm × 5 mm were cut from the same location of the produced midsoles; their masses (M_0_) were determined using an analytical balance after conditioning for 24 h. Then, the samples were soaked in distilled water for 24 h, removed from the water and wiped with drying paper to remove excess water, and their masses (M_1_) were measured on an analytical balance within 2 min. Mass water absorption (M) was calculated using the equation below.

% M = (M_1_ − M_0_)/M_0_ × 100 (2)

where M [g]: mass water absorption, M_0_ [g]: initial mass of the specimens, and M_1_ [g]: mass after 24 h in the water.

#### 2.3.6. Tensile Test

**Figure 1:**
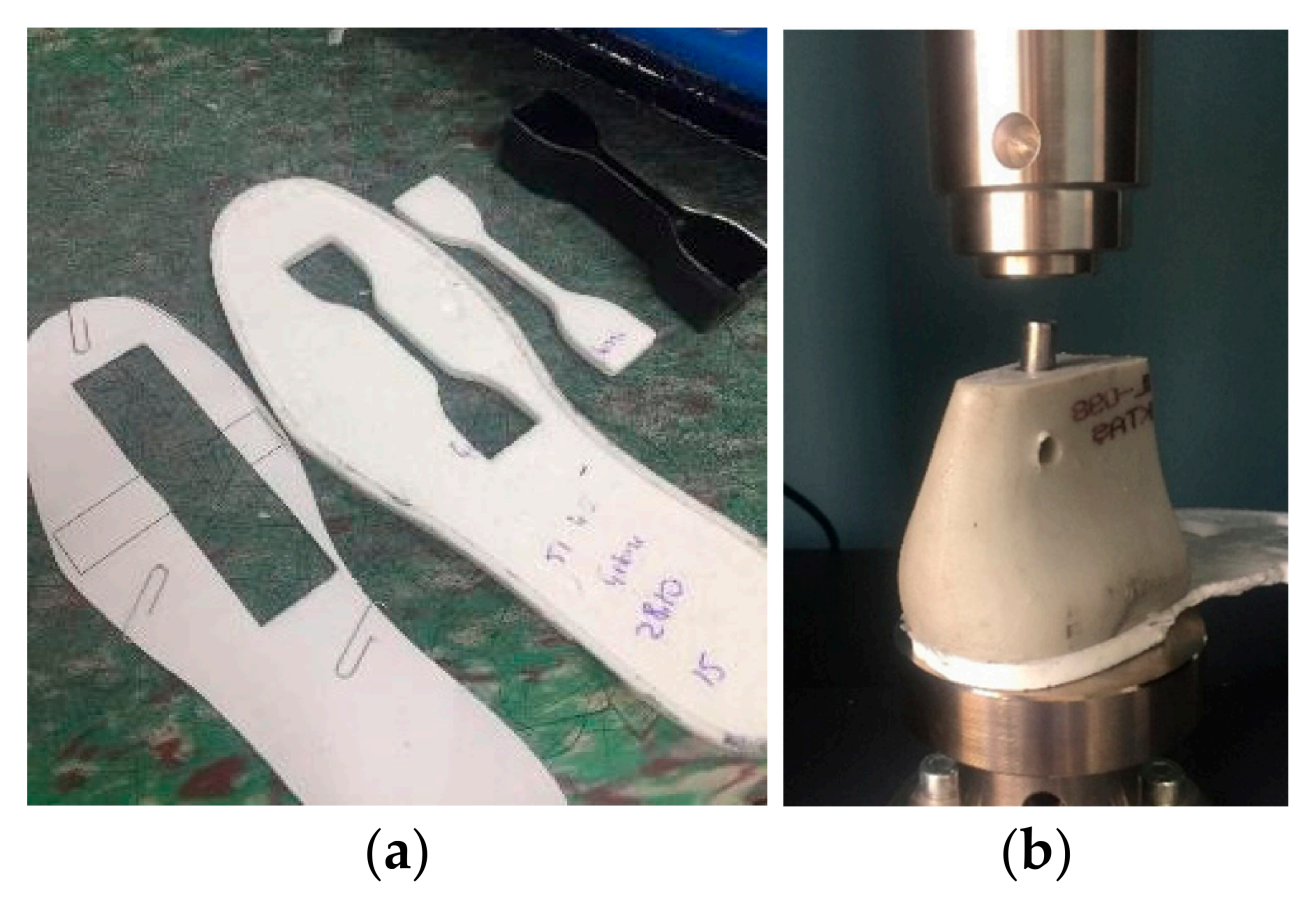
Preparation and testing: (**a**) location of the tensile test specimen; (**b**) compression test setup.

Three tensile test specimens were taken from the same location of the produced midsoles (Figure 1a). The test specimens were prepared in accordance with TS EN ISO 17709 [26], and the tensile test was carried out according to TS EN 12803 [27]. The test was performed at room temperature using a universal testing machine (Shimadzu AGS-X, Kyoto, Japan) equipped with a 50 kN load cell at a crosshead speed of 80 mm/min.

#### 2.3.7. Compression Test

Compression test on three samples was performed in accordance with TS EN 12743 [28] standard using a universal testing machine (Shimadzu AGS-X, Kyoto, Japan). To obtain a homogeneous load distribution in the base region of the specimen, the device shown in Figure 1b was used. Accordingly, the sample to be tested was placed on the compression plate, and a polymer apparatus was used to obtain homogeneous pressure on the sample.

#### 2.3.8. Wear Test

Wear test specimens were prepared for three samples according to TS EN ISO 17709 [26] standard using a cutting mold with a 16 ± 0.2 mm diameter hollow. The wear test of the obtained specimens was performed according to TS EN 12770 [29] standard. The samples were subjected to the test using a wear test machine (HD-P307, Haida International Equipment, Guangdong, China) with a 60-grit abrasive paper, traveling 40 m at 42 rpm under a force of 7.5 ± 0.1 N. However, since the wear on the samples was completed before the end of the travel distance, half the distance (20 m) was used, and the results were multiplied by 2 as specified in the standard. This correction was used only to report the standard-corrected abrasion mass loss under the applied TS EN 12770 test conditions and was not interpreted as evidence of a linear wear-rate relationship for the foam materials. The corrected mass-loss values were subsequently normalized to the initial specimen mass and reported as wt.% for comparison among formulations. The equation used for the determination of the standard-corrected mass loss is shown below.

M = m × S_0_/S (3)

where M: relative mass loss (mg), m: mass loss (mg), S_0_: nominal mass loss of standard rubber (200 mg), and S: average mass loss of standard rubber (mg).

#### 2.3.9. Dry and Wet Tribometric Test

Dry and wet tribometric tests were performed to evaluate the frictional response of polyurethane samples under reciprocating sliding conditions. The tests were carried out in linear mode using a sinusoidal motion profile with a full amplitude of 6.00 mm, maximum linear speed of 1.50 cm/s, frequency of 0.80 Hz, normal load of 1.00 N, and data acquisition rate of 20 Hz. Each test was continued until a total sliding distance of 10.00 m was reached or until the coefficient of friction exceeded µ = 2.00 (TRB3, Anton Paar Gmhb, Graz, Austria). Dry tests were conducted under ambient conditions without lubricant. For wet tests, ultrapure water was used as the lubricant, and 0.10 mL of water was applied to the contact interface before testing. The coefficient of friction was recorded as a function of sliding distance. These measurements were used to compare material-surface COF under controlled laboratory contact conditions and were not intended to represent complete outsole traction performance.

#### 2.3.10. Temperature-Dependent Flexural Resistance Test

**Figure 2:**
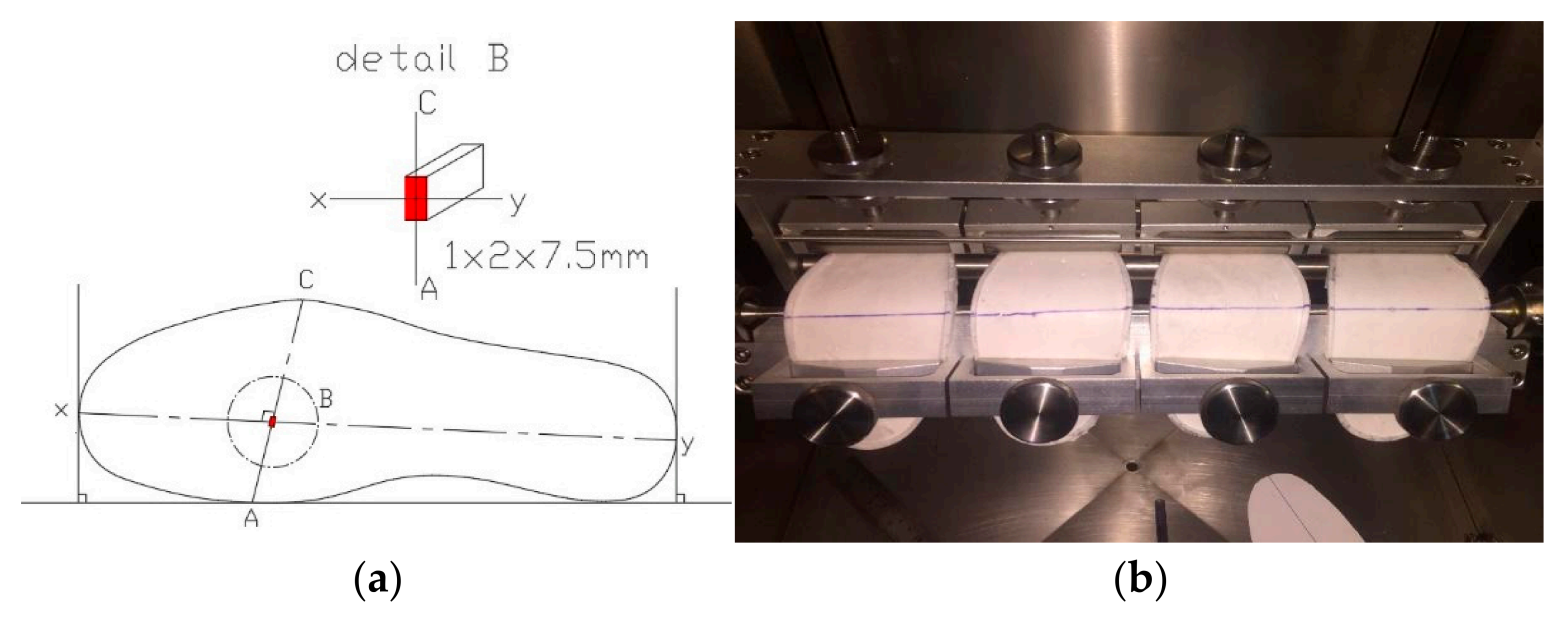
Description of the temperature dependent flexural resistance test: (**a**) location of the incision on the test sample; (**b**) flexural resistance test setup with mounted samples.

The flexural resistance test was conducted on three samples according to TS EN ISO 17707 [30] using a flexural testing machine (HD-E702-150B40, Haida International Equipment, Guangdong, China). Test specimens were incised along the flexing line (CA), using the intersection point of the longitudinal axis (XY) and the flexing line (CA) as the center of the incision. The incision length was 2 ± 0.1 mm, and the incision depth was equal to the midsole thickness (Figure 2a). The specimens were tested for 5 h (30,000 cycles) at −20 °C, 0 °C, 20 °C and 40 °C with 100 bending movements per minute (Figure 2b). After testing, the increase in incision length was measured.

## 3. Results and Discussion

In the present study, the mechanical and physical properties of polyurethane (PU) midsoles prepared using different PB/IM ratios and corresponding isocyanate indices were systematically investigated. A laboratory-scale aluminum mold equipped with a cooling system was designed, and a controlled PU midsole production setup was established. Preliminary trials were conducted over a wide range of formulation conditions to identify compositions yielding structurally stable and fully reacted polyurethane foams. The low-index formulations (93 and 105) showed incomplete bonding and/or dimensional instability under the selected processing conditions; therefore, these specimens were retained as preliminary processing observations rather than used for full characterization.

**Figure 3:**
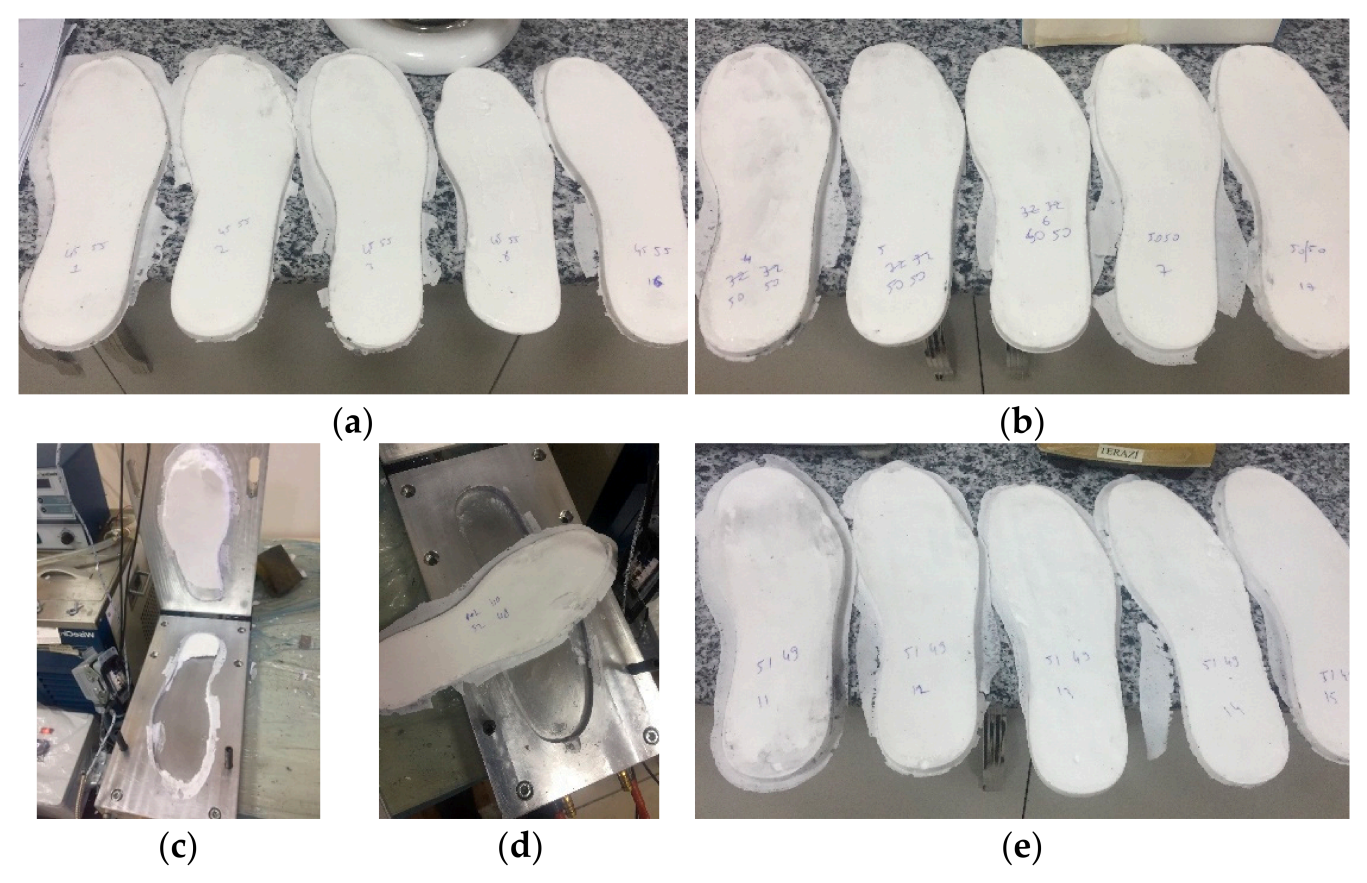
Representative polyurethane samples produced at different isocyanate indices: (**a**) 138, (**b**) 113, (**c**) 93, (**d**) 105, and (**e**) 110.

Based on these observations, formulations corresponding to isocyanate indices of 138, 113, and 110 were selected for detailed investigation because they provided stable foam structures suitable for mechanical, thermal, and microstructural characterization. Representative photographs of the produced PU midsoles, including the preliminary low-index trials, are presented in Figure 3. The results discussed in the following sections focus on the three stable formulations to enable a consistent comparison of structure-property relationships.

The results obtained for the three stable formulations indicate that the measured properties did not change uniformly across the investigated formulation range. Although differences in tensile strength, abrasion behavior, and thermal response were observed, the compressive response showed comparatively limited variation under the applied test conditions. Considering that changes in the PB/IM ratio simultaneously modified the isocyanate index, formulation composition, density, and cellular morphology, these findings are interpreted as formulation-specific trends. Among the investigated formulations, the 113-index formulation exhibited the most balanced combination of the evaluated properties.

### 3.1. FTIR

**Figure 4:**
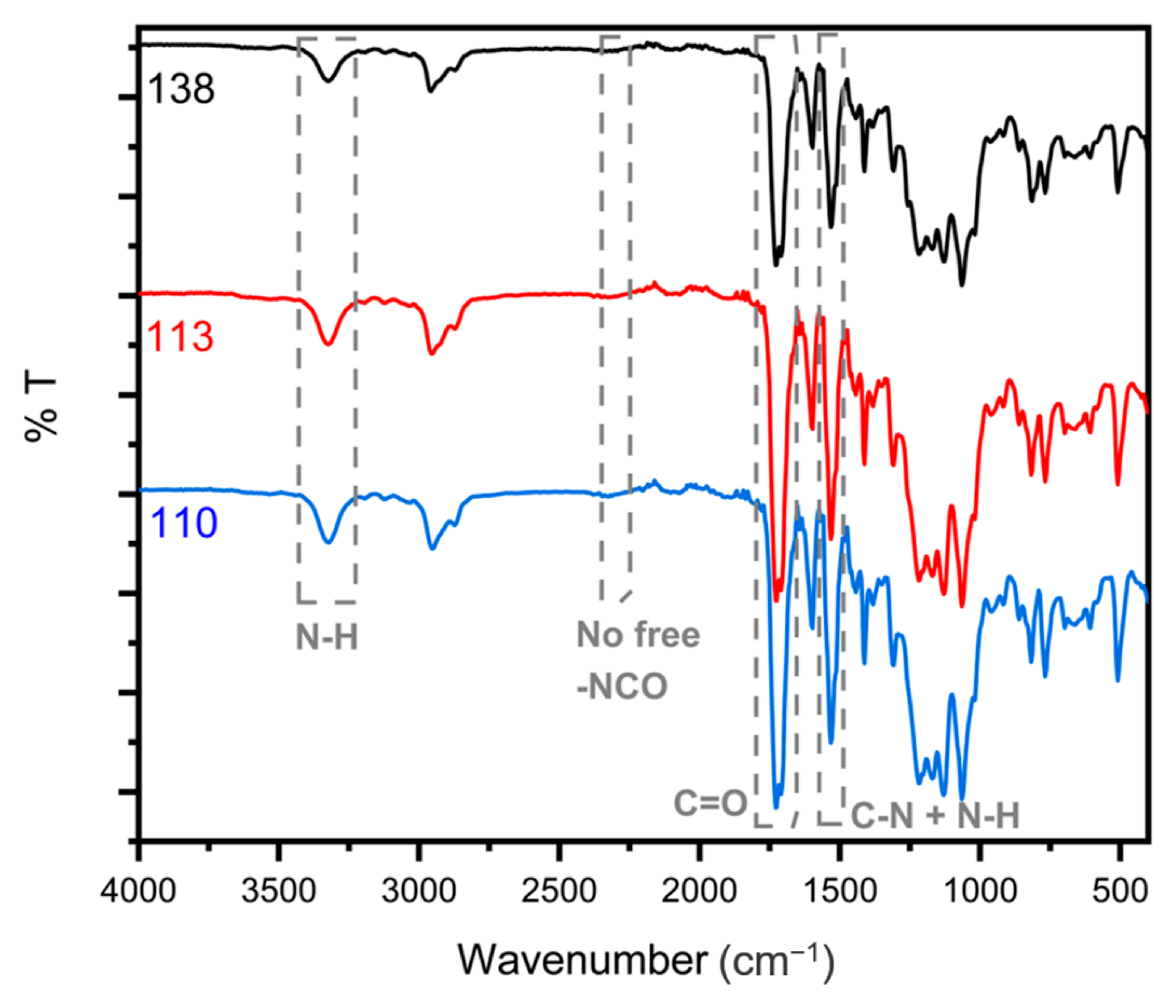
FTIR spectra of polyurethane samples showing the effect of isocyanate index on chemical structure: isocyanate indices of 110, 113, and 138.

Figure 4 presents the FTIR spectra of the polyurethane samples synthesized using different isocyanate indices. The characteristic absorption bands observed in all samples confirm the successful formation of a urethane-based polymer network. In particular, the broad band observed at 3321 cm^−1^ was assigned to N–H stretching vibrations in urethane and/or urea groups, indicating the presence of hydrogen-bonded hard segments within the polymer matrix.

The carbonyl (C=O) stretching vibrations, attributed to the ester carbonyls of the polyester-based soft segments and urethane groups, appeared as a strong absorption peak around 1728 cm^−1^. Additionally, the amide II band detected around 1527 cm^−1^ was attributed to N–H bending and C–N stretching vibrations. These bands support the formation of polyurethane morphology and urethane/urea-related linkages.

Furthermore, the bands observed in the 1130–1219 cm^−1^ range were associated with C–O–C, C–O, and C–N stretching vibrations specific to urethane linkages and polyester polyol chains. The C–H stretching and bending vibrations belonging to the aliphatic methylene groups in the polymer backbone were detected at 2955 cm^−1^ and 1410–1440 cm^−1^, respectively.

The absence of an absorption peak in the 2250–2270 cm^−1^ region, which is assigned to unreacted free isocyanate (–NCO) groups, indicates that no detectable free isocyanate groups remained in the analyzed samples. The overall similarity of the FTIR spectral profiles shows that variations in the isocyanate index did not change the fundamental polyurethane skeleton. However, relative intensity variations in the N–H, C=O, amide II, and C–O–C/C–N bands suggest differences in hard-segment content, hydrogen bonding, and phase organization. Within this context, without overinterpretation, the 138-index sample suggests a denser hard-segment network, whereas the 113-index formulation appears to provide a more balanced hard/soft segment arrangement, consistent with its overall mechanical performance and durability.

### 3.2. Thermal Analysis

#### 3.2.1. DSC

**Figure 5:**
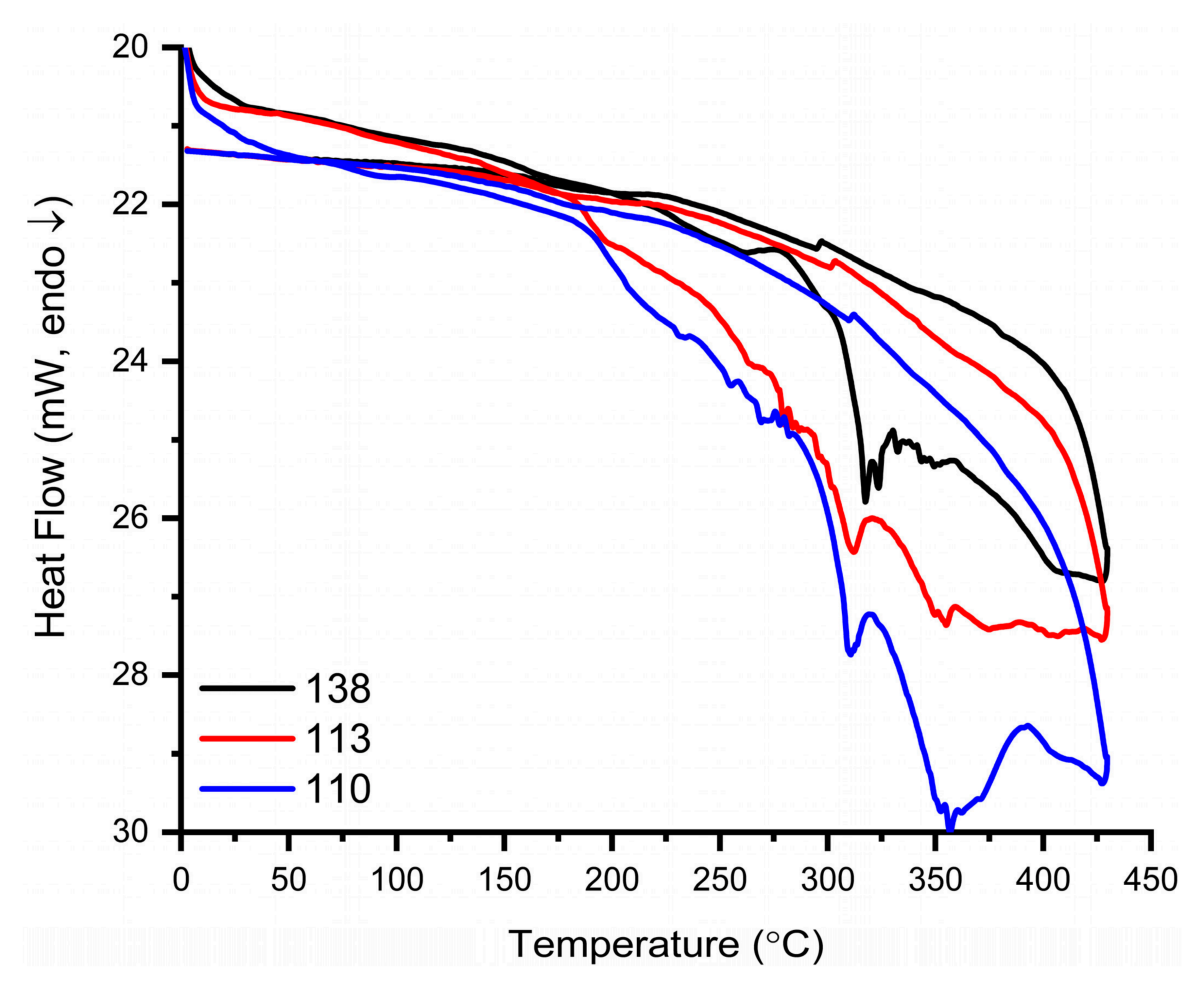
DSC thermograms of polyurethane samples showing the effect of isocyanate indicies 138, 113, and 110.

Figure 5 presents DSC thermograms of polyurethane samples prepared at isocyanate indices of 138, 113, and 110. Since the curves were not vertically shifted, the observed heat-flow differences reflect the relative thermal responses of the samples under the applied DSC conditions. All samples exhibited broad heat-flow events rather than sharp melting transitions, which is typical of segmented polyurethane systems composed of polyester-based soft segments and urethane/urea-rich hard domains. However, because the DSC scan was extended to 450 °C and this temperature range overlaps with the onset of polyurethane degradation, the high-temperature heat-flow events were interpreted cautiously and discussed together with the corresponding TGA/DTG results.

The 138-index sample showed a relatively delayed heat-flow response up to approximately 300 °C, followed by a more pronounced high-temperature event. This behavior may be associated with differences in network rigidity and hard-segment contribution; however, these assignments should be considered qualitative because thermal degradation may also contribute to the heat-flow response in this temperature region. In contrast, the 113- and 110-index samples displayed broader and more gradual heat-flow changes, suggesting differences in the relative contributions of soft and hard segments under the applied heating conditions.

Overall, the DSC results indicate differences in the heat-flow responses of the investigated polyurethane foam formulations under the applied heating conditions. Nevertheless, DSC was not used alone to draw definitive conclusions regarding thermal degradation or the original segmental organization at high temperatures. Instead, high-temperature DSC events were interpreted together with the corresponding TGA/DTG data, and the discussion of thermal degradation and high-temperature mass-retention behavior was based primarily on TGA/DTG analysis.

#### 3.2.2. TGA/DTG

**Figure 6:**
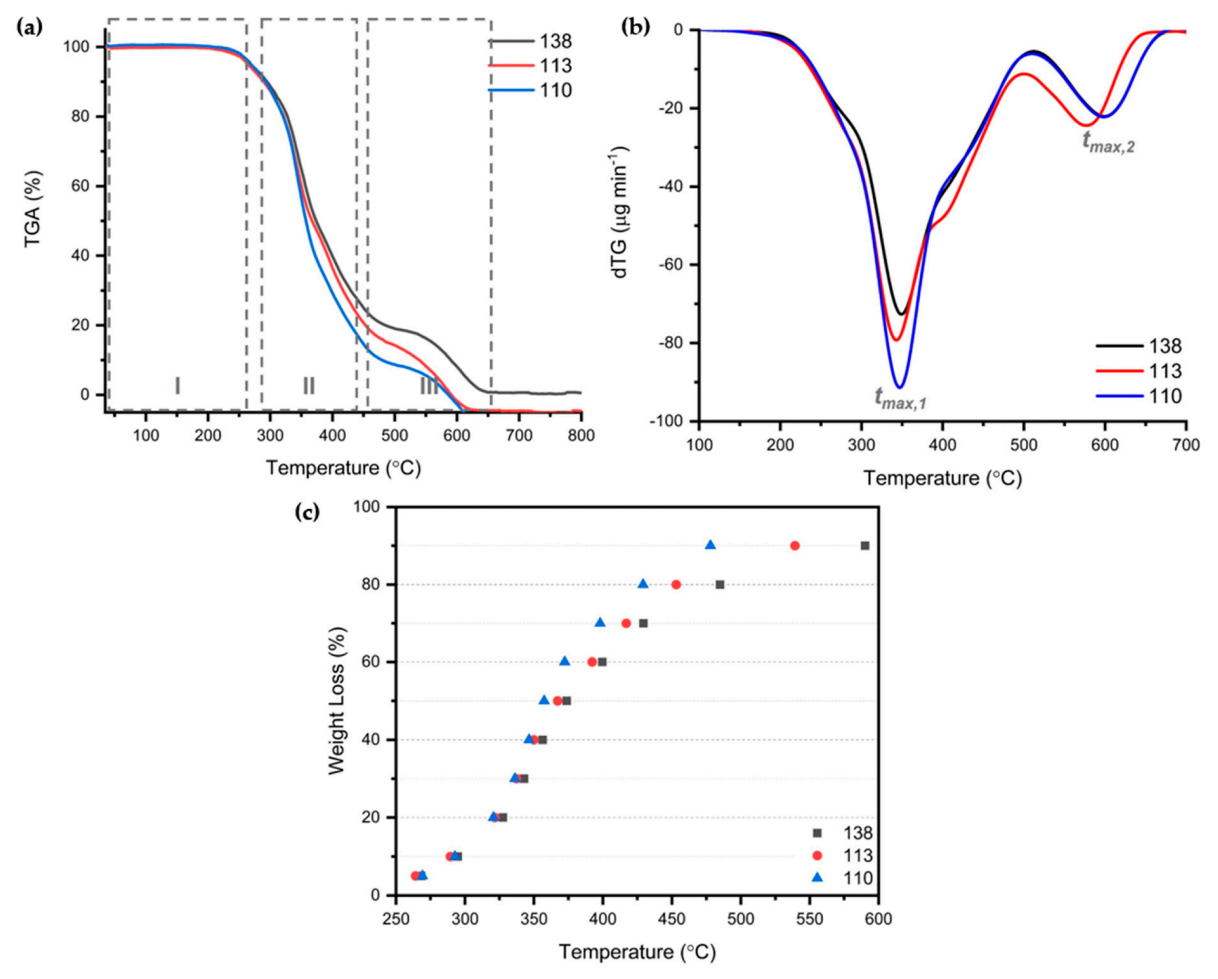
Thermal degradation behavior of polyurethane samples prepared at different isocyanate indices: (**a**) TGA curves; (**b**) DTG curves with characteristic degradation regions; and (**c**) weight losses vs. temperatures.

Figure 6 presents the TGA and DTG profiles of polyurethane samples prepared at different isocyanate indices, while Table 2 summarizes the characteristic degradation temperatures obtained from these analyses. All formulations exhibited a multi-stage thermal degradation behavior, which is characteristic of segmented polyurethane structures.

In the initial degradation region, the temperatures corresponding to 5% and 10% weight loss *t*_5%_ and *t*_10%_ were found to be close to each other among the samples. This indicates that, within the investigated formulation range, the isocyanate index did not cause a pronounced change in the initial thermal stability of the materials. The main degradation stage occurred approximately in the range of 300–430 °C. The first maximum degradation rate temperature *t_max_*_,1_ was determined within a narrow range of 343.2–348.7 °C for all samples. This result indicates that the main degradation stage, associated with the decomposition of urethane/urea linkages and hard-segment-rich domains, proceeds through a similar mechanism in all formulations.

In contrast, the temperatures corresponding to 90% weight loss *t*_90%_ varied significantly depending on the isocyanate index. The sample with an isocyanate index of 138 reached the *t*_90%_ value at 590.1 °C, whereas this value decreased to 539.3 and 478.0 °C for the samples with isocyanate indices of 113 and 110, respectively. This finding indicates that the formulation with the highest investigated isocyanate index exhibited greater mass retention at elevated temperatures. Because several formulation characteristics changed simultaneously, this observation should be interpreted as a formulation-specific trend rather than the isolated effect of the isocyanate index. The second maximum degradation rate temperature *t_max_*_,2_ was determined as 600.0 °C for the samples with isocyanate indices of 138 and 110, and as 577.1 °C for the sample with an isocyanate index of 113. However, considering the lower *t*_90%_ value of the 110-index sample, its high *t_max_*_,2_ value does not indicate superior overall thermal stability; rather, it reflects the degradation behavior of the residual structures remaining at high temperatures.

In summary, the DSC and TGA/DTG findings are complementary. Although the initial and main degradation characteristics were similar among the formulations, the 138-index formulation exhibited improved high-temperature mass retention, as reflected by its higher *t*_90%_ value.

### 3.3. Density

**Figure 7:**
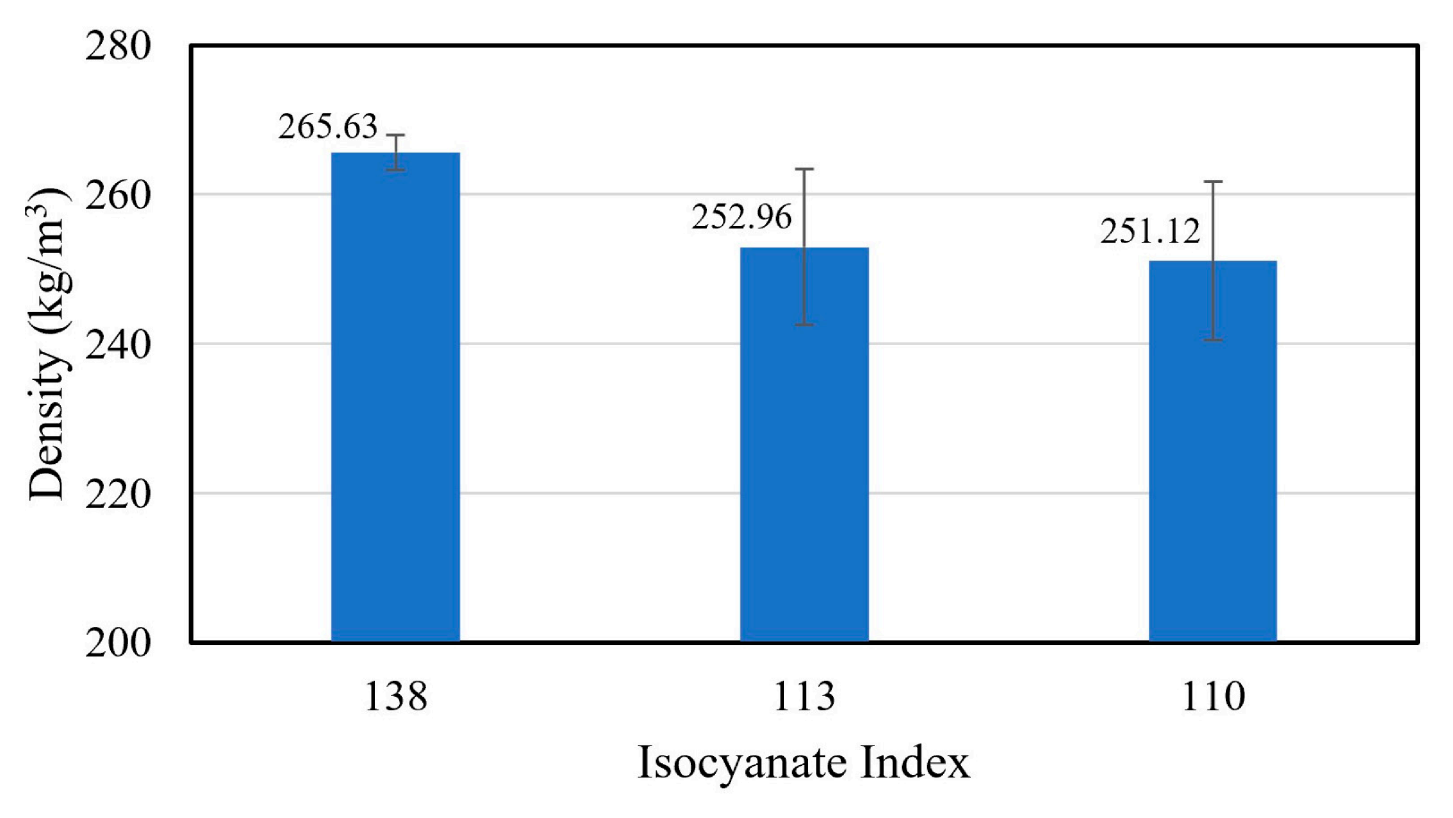
Variation of density with isocyanate index for polyurethane samples.

The effect of isocyanate index on the density of the produced polyurethane midsoles is illustrated in Figure 7. The density of the sample prepared at an isocyanate index of 138 was approximately 5.0% and 5.8% higher than those of the samples prepared at isocyanate indices of 113 and 110, respectively. The minor difference observed between the 113- and 110-index samples can be attributed to the close proximity of their isocyanate indices.

As shown in Figure 7, the 138-index formulation exhibited a higher midsole density than the 113- and 110-index formulations. This trend is consistent with formulation-dependent differences in network development and foam expansion, which arose from the simultaneous changes in PB/IM ratio, isocyanate index, and formulation composition. Since low density is desirable for user comfort, the 113- and 110-index formulations show a weight advantage compared with the 138-index formulation.

The density measurements are consistent with the literature. The polyol/isocyanate ratio has been reported to play an important role in determining PU foam density; formulations with higher isocyanate index can produce smaller cells and a higher cell-wall fraction, resulting in increased foam density [31,32].

### 3.4. SEM Analysis

**Figure 8:**
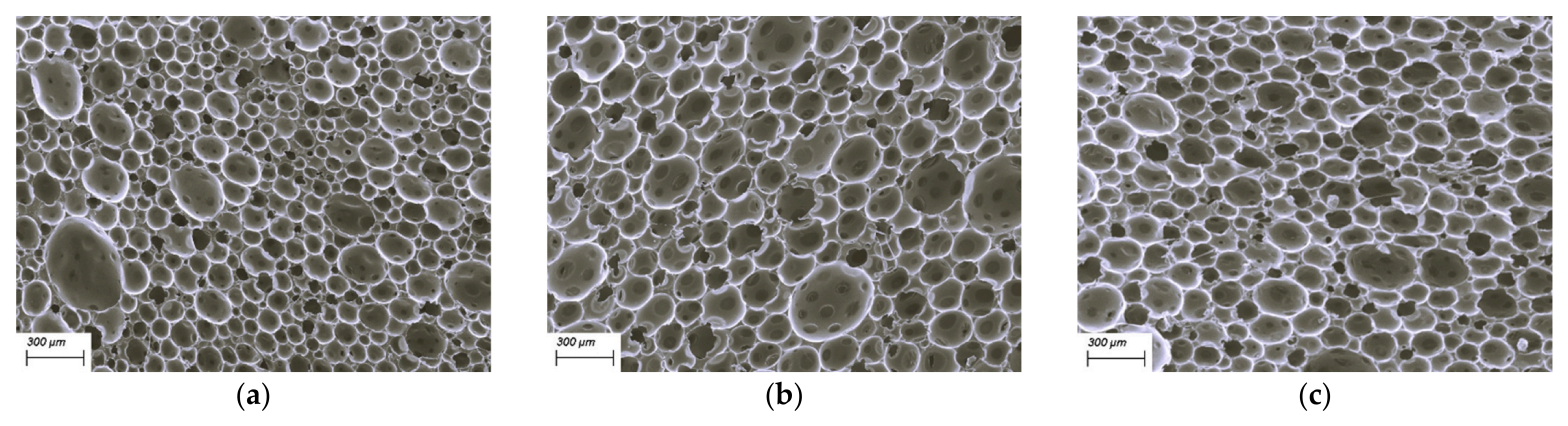
SEM images of polyurethane midsoles prepared at different isocyanate indices: (**a**) 138; (**b**) 113; and (**c**) 110.

SEM images (Figure 8) qualitatively show that the produced midsoles exhibited a partially open cellular morphology regardless of the isocyanate index. The 138-index formulation exhibited thinner cell walls and partial interconnection between adjacent cells than the 113- and 110-index formulations. Such microstructural features can facilitate localized fluid penetration through micro-defects and cut surfaces, which may explain the relatively high water absorption despite the absence of a fully open-cell morphology. The 138-index sample contained predominantly small and relatively uniform cells together with a limited number of large pores, whereas lower-index samples showed a comparatively higher proportion of medium and large pores, indicating a more expanded cellular morphology. This observation is consistent with the microstructural trends reported by Linul et al. [22]. The 113- and 110-index samples exhibited similar pore morphologies, consistent with their comparable density values.

Overall, the SEM observations demonstrate qualitative differences in cellular morphology among the investigated formulations. Because changes in the PB/IM ratio simultaneously affected the isocyanate index, formulation composition, and foaming behavior, these morphological differences should not be attributed solely to the isocyanate index. Quantitative image analysis of cell size distribution and cell density would be required to establish direct structure–property relationships. Our qualitative observations are supported by Dworakowska et al. [33], who reported that increasing the isocyanate ratio in polyurethane samples prepared from polyol and isocyanate mixtures reduced cell size and produced a more homogeneous cell structure.

### 3.5. Water Absorption Test

**Figure 9:**
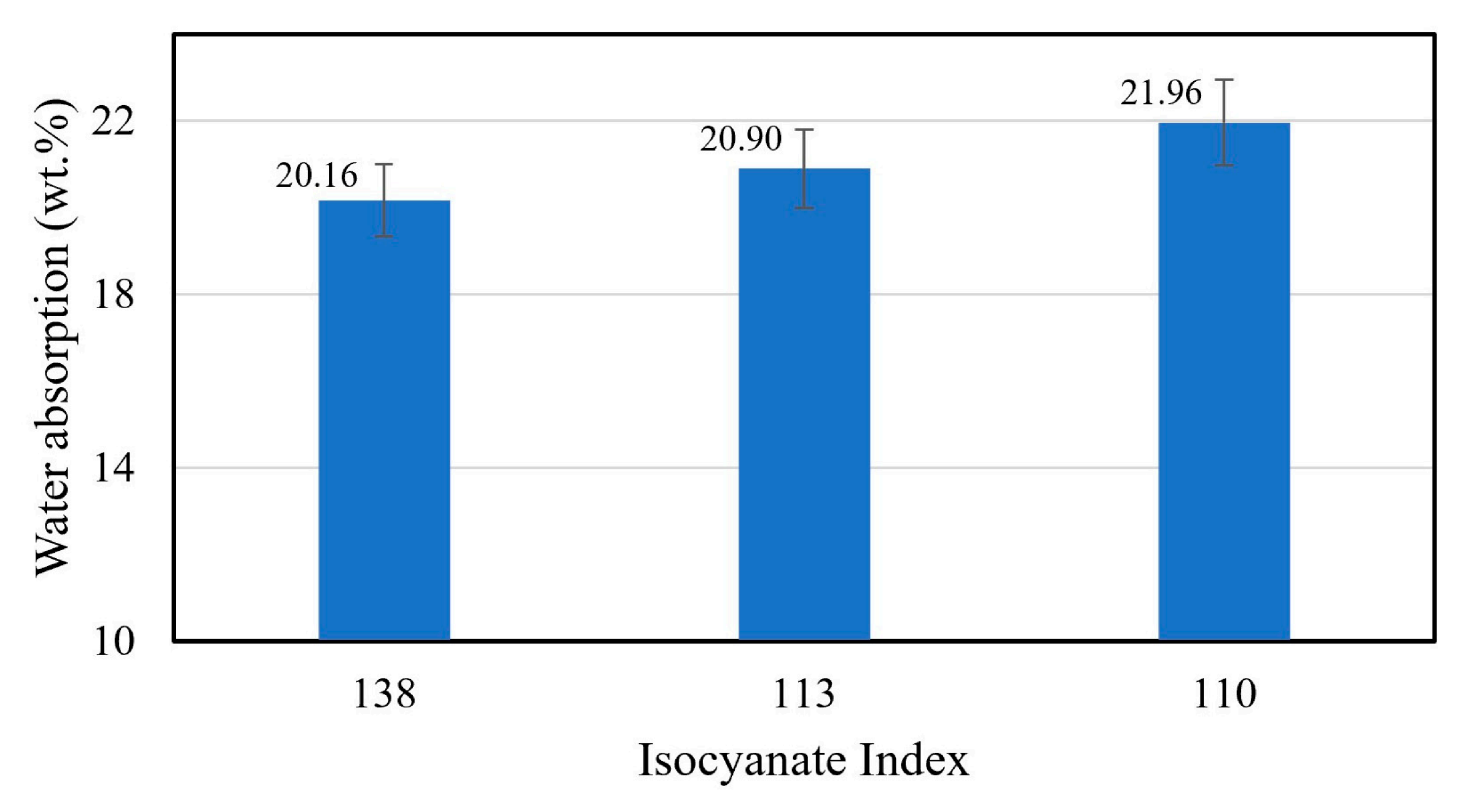
Water absorption of polyurethane samples as a function of isocyanate index.

Water absorption tests were conducted on samples with different polyol/isocyanate contents by determining water uptake after immersion in distilled water for 24 h. Water absorption increased slightly from 20.16 to 21.96 wt.% as the isocyanate index decreased from 138 to 110, respectively (Figure 9). Although higher polyol content may increase open-cell content and porosity, this trend was not clearly resolved in the qualitative SEM images. Therefore, the water-absorption behavior is attributed to the combined effects of cellular morphology, cut-surface exposure, and network polarity. The observed increase in water absorption with increasing relative polyol content is consistent with the literature.

The isocyanate index (NCO/OH ratio) significantly influences both the crosslink density and the stability of reaction products formed during polyurethane foaming reactions. In polyurethane systems employing water- and catalyst-containing polyol formulations, urethane bonds are formed through the reaction of isocyanate groups with hydroxyl functionalities, while urea bonds originate from the reaction between isocyanate and water present in the formulation. An increase in the isocyanate index promotes higher crosslink density and rigid segment formation, thereby reducing chain mobility and the free volume available for water diffusion. Higher isocyanate indices are associated with improved cell integrity and reduced open porosity, leading to decreased water absorption in rigid polyurethane foams [34]. Moreover, an adequate isocyanate index (NCO/OH ≥ ~1) facilitates more complete reactions involving polyols, water, and catalysts, resulting in a more homogeneous cellular structure and limiting the formation of macroporous pathways that enable water penetration [34,35].

### 3.6. Tensile Test

**Figure 10:**
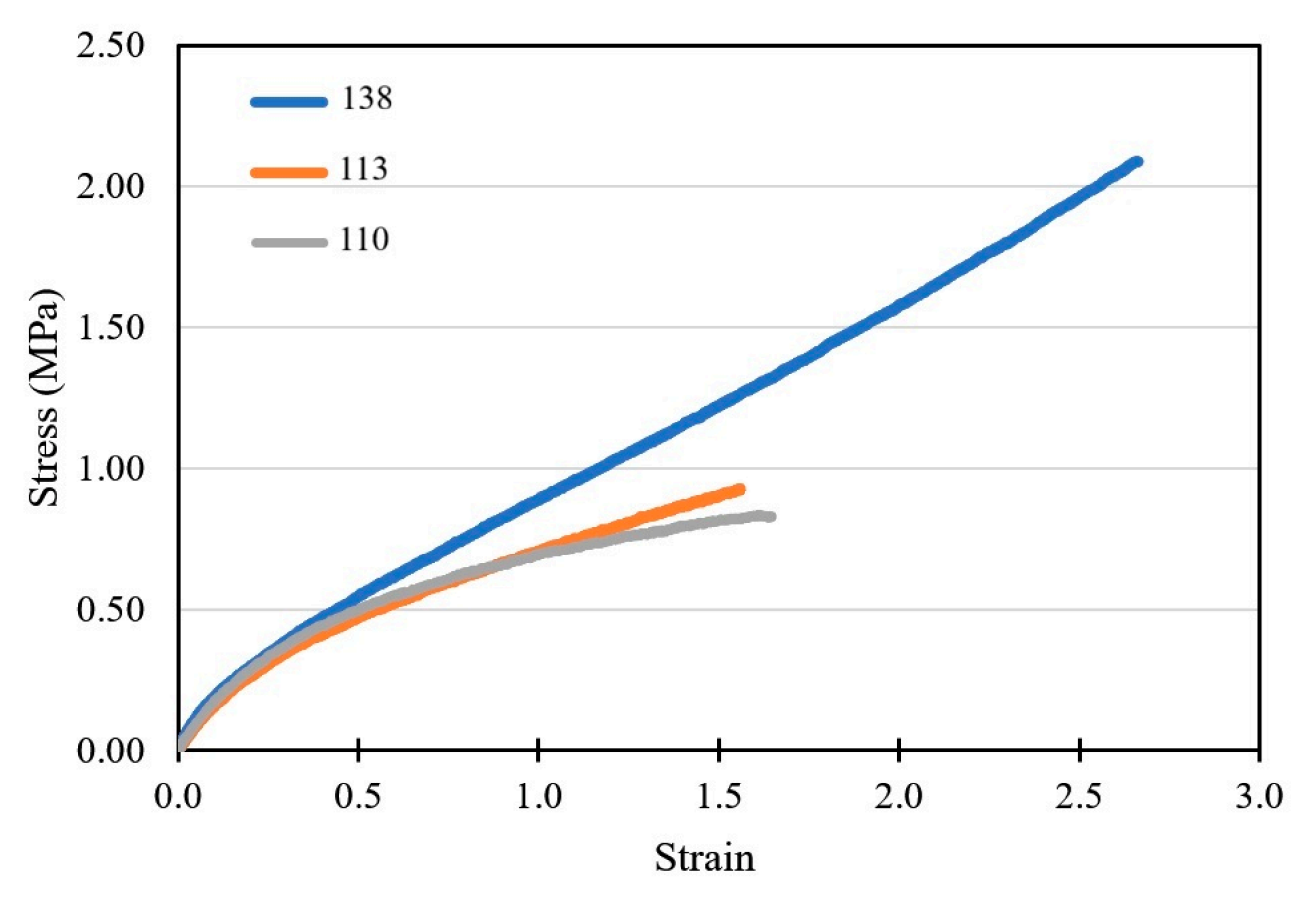
Tensile stress–strain curves of polyurethane samples prepared at different isocyanate indices.

Tensile stress–strain curves of samples prepared at isocyanate indices of 138, 113, and 110 are presented in Figure 10. The tensile tests showed that the 138-index formulation exhibited a higher strength at break than the 113- and 110-index formulations. As shown in Figure 10, the tensile strength at break values were 0.83 ± 0.10, 0.92 ± 0.11, and 2.09 ± 0.35 MPa for the 110-, 113-, and 138-index samples, respectively. This result is consistent with the literature [36].

Isocyanate type and stoichiometry directly affect hard-segment content, crosslink density, and the balance between urethane and other reaction products; these parameters determine the tensile strength and elastic modulus of polyurethanes. Increasing the hard-segment fraction or crosslink density, for example by increasing the isocyanate index or using higher-functional isocyanates, generally increases tensile strength and modulus [37]. Studies on segmented and block copolymer systems show that hard-segment content plays a key role in tensile performance, particularly by increasing tensile strength and modulus depending on the degree of phase organization [38]. Furthermore, modifications in crosslink chemistry and density have been reported to improve the tensile performance and elastic response of polyurethane-based elastomers, confirming the importance of network structure in mechanical behavior [39]. In the present study, the 138-index sample exhibited the highest tensile strength and sustained higher strain before failure compared with the lower-index formulations. Accordingly, the tensile response reflects the collective characteristics of the investigated formulations and cannot be attributed to the isocyanate index alone.

### 3.7. Compression Testing

**Figure 11:**
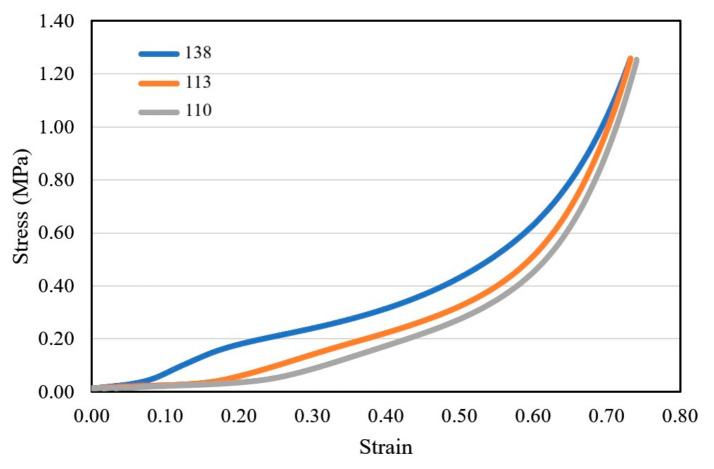
Compressive stress-strain curves of polyurethane samples prepared at different isocyanate indices.

Figure 11 presents the compressive stress-strain behavior of the polyurethane samples at room temperature. All formulations exhibited a typical elastomeric foam response, consisting of an initial linear elastic region followed by a plateau/densification region associated with cell-wall bending and collapse. Under the applied standard compression test conditions, the compressive responses of the samples were relatively close, with all formulations reaching similar stress levels at the maximum applied deformation/load. However, the 138-index sample showed a slightly higher stress response over most of the strain range, indicating that the effect of isocyanate index on compression behavior was less pronounced than its effect on tensile behavior.

Increasing the NCO/OH ratio generally increases the compressive strength and elastic modulus of PU foams by increasing crosslink density and hard-segment content. Yeoh et al. [40] observed improved compressive stress at higher isocyanate ratios and attributed this improvement to increased crosslink density in the hard segments and stronger intermolecular interactions. Similarly, Liao et al. [41] reported increased specific compressive strength when the polyol reacted with isocyanate at twice the molar ratio, attributing this behavior to enhanced crosslink formation and hard-segment development. These findings are consistent with the general polyurethane structure–property relationship, in which higher hard-segment content and a denser crosslinked structure can create a tougher and more compression-resistant network [40,41]. In the present study, the standard compression test configuration and constant maximum load limited the separation among the formulations; so, the compression results should be interpreted comparatively rather than as a complete description of cyclic midsole cushioning behavior.

### 3.8. Wear Test

#### 3.8.1. Standard Abrasion Wear Test

**Figure 12:**
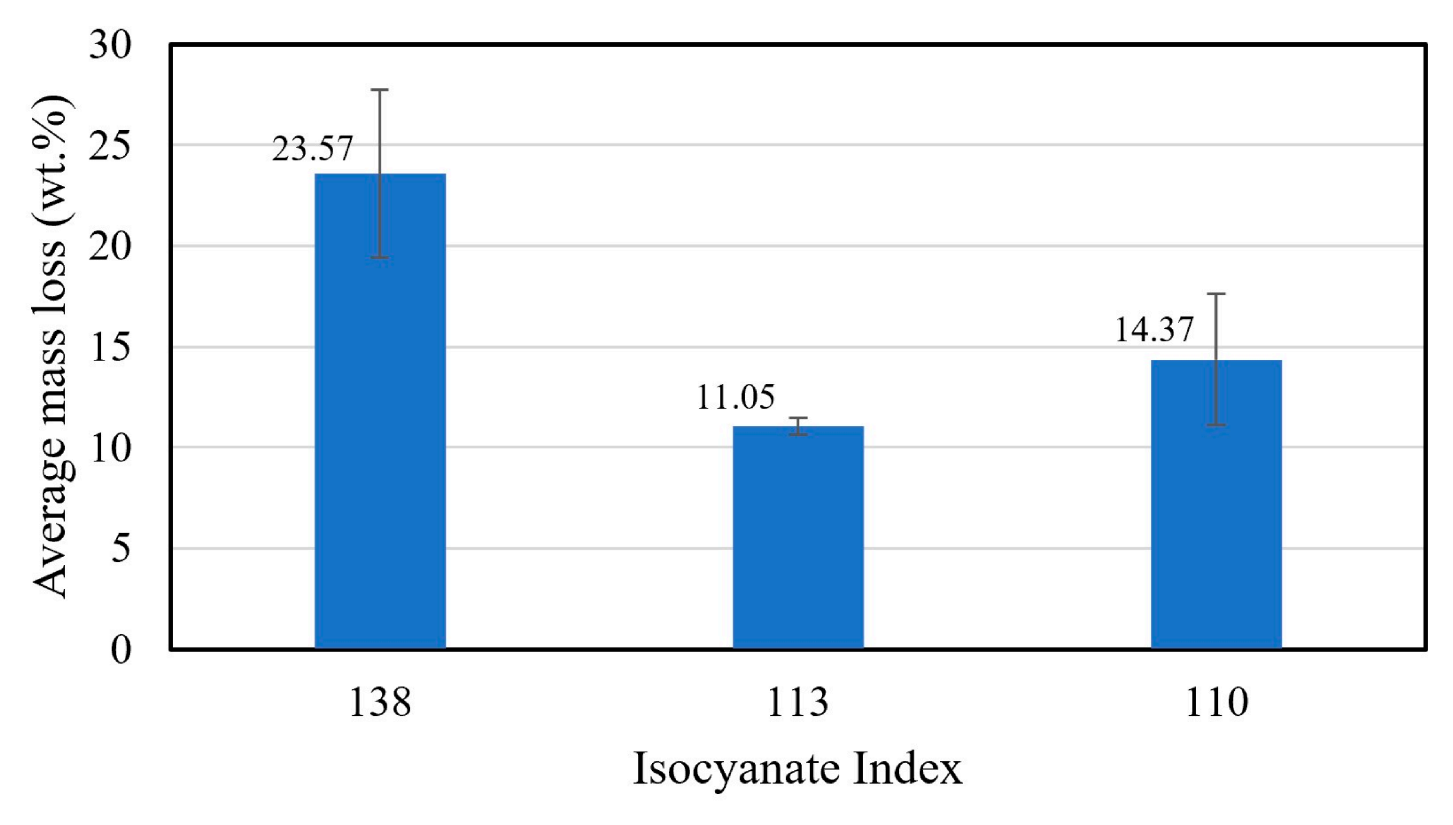
Average mass loss of polyurethane samples as a function of isocyanate index.

The average values and standard deviations obtained from the abrasion wear test are presented in Figure 12. The highest average mass loss was observed for the 138-index sample, with a value of 23.57 wt.%, whereas the lowest average mass loss was recorded for the 113-index sample, with a value of 11.05 wt.%. Compared with the 113-index formulation, both higher and lower isocyanate indices resulted in increased mass loss, indicating a non-monotonic wear response.

Isocyanate type and index (NCO/OH ratio) influence wear behavior by altering hard-segment content, hydrogen bonding, and crosslink density. Materials with higher isocyanate-derived hard-segment content or hard-segment-/urea-rich structures may exhibit harder surfaces; however, excessive rigidity can also promote brittle microfracture and material removal during abrasion. Domańska et al. [42] and other studies have shown that the isocyanate chemistry affects chain packing and mechanical strength, both of which are important for wear resistance [42,43]. Although increasing isocyanate index is generally associated with enhanced hardness and strength, the present results show that abrasion behavior does not follow a simple monotonic relationship with isocyanate index. As shown in Figure 12, the 138-index sample had the highest mass loss under the applied test conditions, whereas the 113-index sample showed the lowest mass loss. These results indicate that, among the investigated formulations, the 113-index formulation exhibited the lowest abrasion mass loss under the applied test conditions. Accordingly, the observed wear performance should be regarded as a formulation-specific response arising from the combined characteristics of the investigated formulations rather than from the isocyanate index alone.

To further clarify the surface-contact behavior of the polyurethane samples, dry and wet tribometric tests were conducted by monitoring the coefficient of friction under sliding conditions.

#### 3.8.2. Dry and Wet Tribometric Friction Behavior

**Figure 13:**
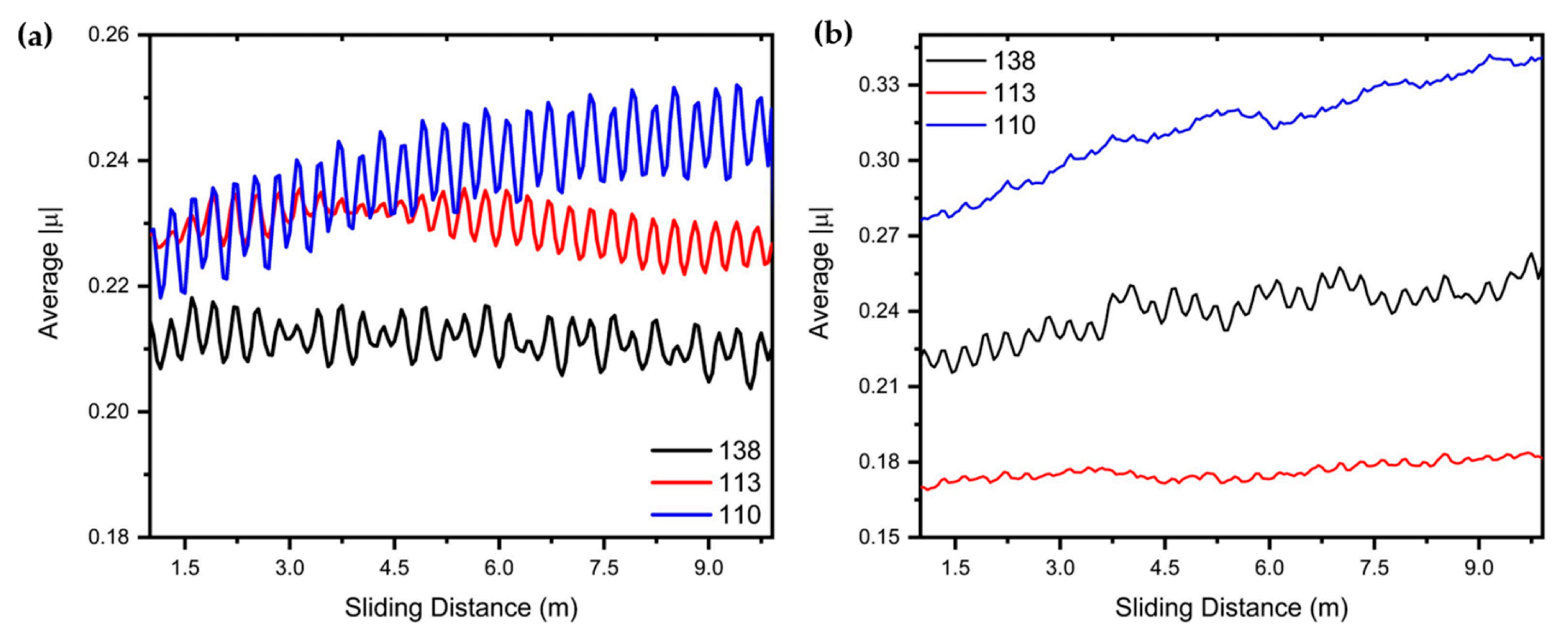
Distance-dependent binned-average absolute coefficient of friction, average ∣μ∣ of polyurethane samples under (**a**) dry and (**b**) wet sliding conditions.

Figure 13 shows the COF behavior of the polyurethane samples under dry and wet sliding conditions, while the corresponding steady-state COF values are summarized in Table 3. The COF curves were plotted using distance-bin-averaged absolute values to improve visual clarity, whereas the steady-state coefficient of friction (μ_ss_) was calculated from the raw absolute COF data over the 1–9.85 m sliding-distance range. These results should be interpreted as controlled material-contact COF values and not as a direct ranking of outsole traction or slip resistance.

Under dry sliding conditions, the 138-index sample exhibited the lowest μ_ss_ value of 0.211, followed by the 113- and 110-index samples with values of 0.230 and 0.238, respectively. The lower COF of the 138-index sample may be associated with its higher hard-segment contribution and more rigid network structure, which can reduce surface deformation during sliding.

In contrast, under wet sliding conditions, the friction behavior changed markedly. The 113-index sample showed the lowest μ_ss_ value of 0.176 under the applied wet test conditions. The 138-index sample exhibited an intermediate μ_ss_ value of 0.240, while the 110-index sample showed the highest value of 0.313. This result suggests that water did not act as an equally effective lubricating medium for all formulations. Instead, the wet COF response appears to depend on the balance between hard-segment rigidity and soft-segment mobility.

Overall, the steady-state COF values indicate that the 138-index formulation exhibited the lowest COF under dry sliding, whereas the 113-index formulation showed the lowest COF under wet sliding. In contrast, the 110-index sample displayed the highest COF in both environments, particularly under wet conditions, suggesting that its lower isocyanate index may increase surface compliance and interfacial resistance during sliding. For footwear applications, these COF results should be considered as one component of surface-contact behavior and should not be used alone to infer complete midsole or outsole performance.

### 3.9. Temperature-Dependent Flexural Resistance Test

To determine the flexural resistance of the midsoles produced from polyurethane samples prepared at isocyanate indices of 138, 113, and 110, the samples were subjected to 30,000 cycles at 100 bending movements per minute at −20 °C, 0 °C, 20 °C, and 40 °C. All specimens, regardless of the isocyanate index, failed at −20 °C. This behavior can be attributed to reduced segmental mobility and increased brittleness of the polyurethane network at sub-zero temperature, which facilitates crack propagation under cyclic bending.

**Figure 14:**
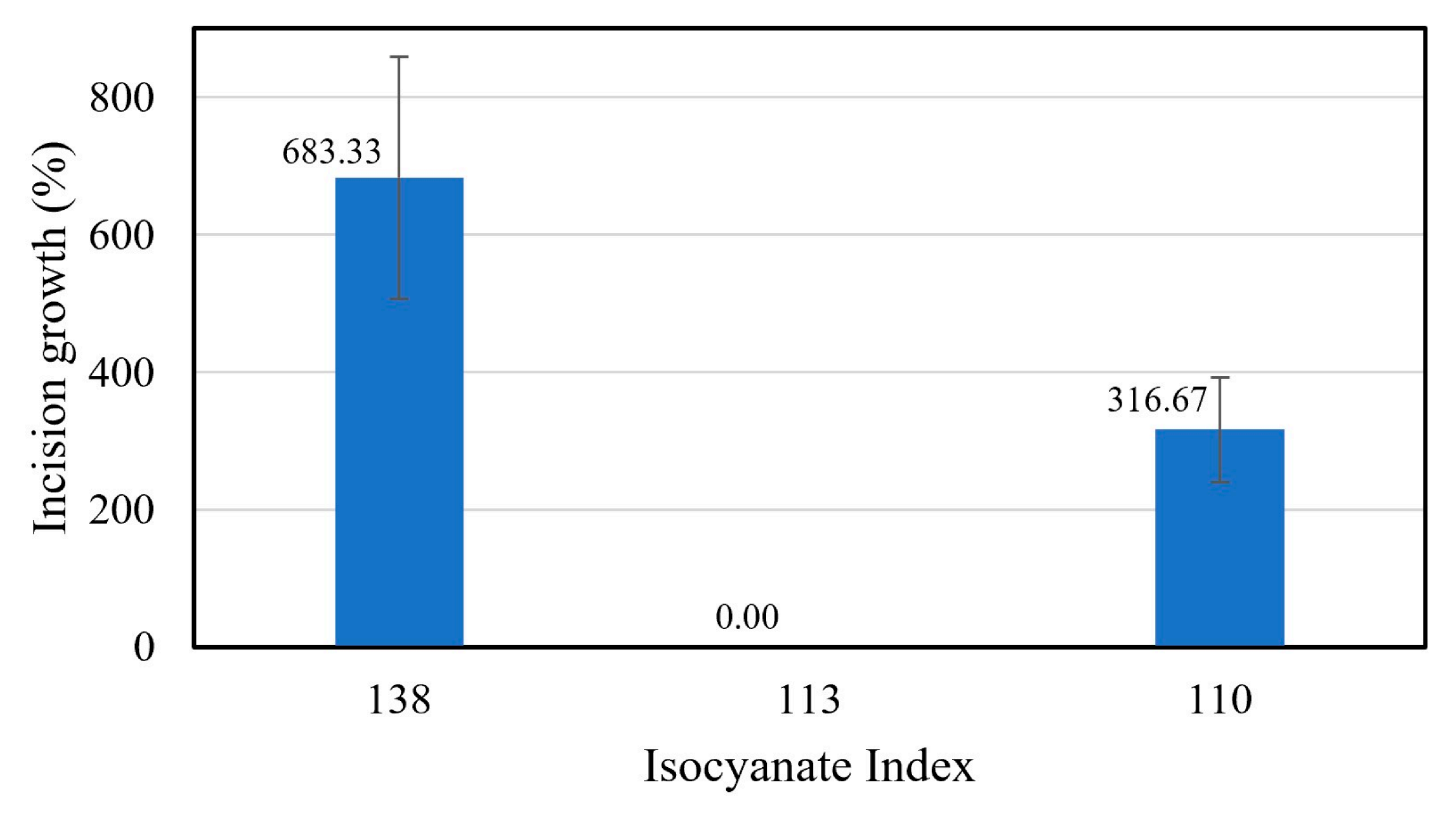
Percent incision growth of polyurethane samples at 0 °C as a function of isocyanate index.

In the flexural resistance test conducted at 0 °C, none of the samples failed completely. However, the 138-index sample showed marked incision growth of 683% (Figure 14). Although the 110-index sample behaved better than the 138-index sample, it also showed considerable incision growth of 317%. In contrast, the 113-index sample showed no measurable incision growth, indicating improved resistance to crack propagation at 0 °C.

Literature indicates that isocyanate index plays an important role in determining hard-segment content, the relative abundance of urethane- and urea-related structures, and the degree of interconnection among hard-segment-rich domains in polyurethane systems. Increasing the isocyanate index generally enhances flexural modulus and initial flexural strength through increased crosslink density and hard-segment fraction. However, excessive hard-segment enrichment may induce brittleness by restricting segmental mobility, thereby facilitating crack initiation and propagation at interfacial regions under cyclic bending. Consequently, bending strain at fracture and fatigue resistance may decrease because the polymer network has reduced energy-dissipation capacity [44,45].

In the flexural resistance test conducted at 20 °C and 40 °C, no incision growth was observed in any of the samples, regardless of the polyol ratio.

## 4. Conclusions

In this study, the chemical structure, thermal behavior, physical properties, tribological response, and mechanical performance of polyester-based polyurethane foams prepared using different PB/IM ratios and corresponding isocyanate indices were systematically investigated for footwear midsole applications. FTIR analyses confirmed the formation of polyurethane networks without detectable free isocyanate groups, while relative band-intensity variations indicated changes in hard-segment-related interactions and hydrogen bonding with changing isocyanate index. DSC and TGA/DTG analyses demonstrated differences in heat-flow response, segmental organization, and high-temperature mass-retention behavior among the investigated formulations. High-temperature DSC events were interpreted together with the corresponding thermal-degradation data.

Physical and mechanical testing showed that the 138-index formulation exhibited higher density and tensile strength than the 113- and 110-index formulations. These differences are consistent with changes in network structure; however, because the PB/IM ratio, isocyanate index, and formulation composition changed simultaneously, they should be interpreted as formulation-specific rather than as the isolated effect of the isocyanate index. However, the compressive stress–strain responses showed relatively limited differences under the applied test conditions, indicating that the effect of isocyanate index on compression behavior was less pronounced than its effect on tensile performance. Water absorption slightly decreased with increasing isocyanate index, which was interpreted in relation to foam morphology, cut-surface exposure, and network polarity.

The wear and COF results revealed that the relationship between isocyanate index and surface-contact performance was not monotonic. The 113-index formulation exhibited the lowest abrasion mass loss, whereas the 138-index sample showed the highest mass loss under the standard abrasion test. Tribometric analyses showed that the 138-index formulation had the lowest steady-state COF under dry sliding conditions, while the 113-index formulation exhibited the lowest COF under wet sliding conditions. These contrasting results indicate an environment-dependent surface-contact response and should not be interpreted as a complete traction or slip-resistance ranking. Instead, they suggest that the sliding response is affected by the balance between hard-segment rigidity, soft-segment mobility, and surface deformation behavior.

Temperature-dependent flexural resistance tests demonstrated that all samples failed under cyclic bending at −20 °C, indicating limited low-temperature flexibility of the investigated PU foam systems. At 0 °C, no incision growth was observed for the 113-index formulation, whereas the 138- and 110-index samples exhibited marked incision propagation. At 20 °C and 40 °C, no incision growth was observed in any sample. These results indicate that, among the investigated formulations, the intermediate (113-index) formulation exhibited the greatest resistance to crack propagation during cyclic bending at 0 °C. This observation should be interpreted within the investigated formulation range rather than as a general effect of the isocyanate index.

Overall, the polyurethane foam prepared at an isocyanate index of 113 exhibited the most balanced combination of tensile performance, abrasion resistance, COF behavior, water absorption, and flexural durability among the stable formulations examined. Within the tested formulation range, the results indicate that careful formulation design is required to achieve a balanced combination of mechanical, tribological, and durability-related properties for footwear midsole applications. Because the full characterization was limited to three stable formulations, the findings apply only to the investigated formulation range and do not define a universal optimum design window. Future work should include a larger number of replicates, formal statistical analysis, quantitative SEM-based cell analysis, cyclic compression/fatigue measurements, rebound resilience, compression set, and aging/hydrolysis tests to strengthen the application-specific validation of polyester-based PU foams for footwear midsoles.

## Figures and Tables

**Table 1:** Stoichiometric formulation parameters and recalculated isocyanate indices of polyurethane samples according to the polyol blend–to–isocyanate mixture (PB/IM) ratios. The hydroxyl contribution of DABCO^®^ 25 S was included in the total OH-equivalent calculation.

PB/IM Ratio % (wt./wt.)	BDO (g)	Water (g)	DABCO 25 S (g)	Polyester Polyol (g)	Total OH (eq)	NCO (eq)	ISO Index
45/55	0.72	0.059	0.060	17.161	0.0408	0.0565	138
50/50	0.80	0.066	0.060	19.074	0.0452	0.0513	113
51/49	0.82	0.067	0.060	19.453	0.0462	0.0506	110
52/48	0.83	0.069	0.060	19.838	0.0470	0.0492	105
55/45	0.88	0.073	0.060	20.987	0.0497	0.0463	93

**Table 2:** Characteristic degradation temperatures of polyurethane samples obtained from TGA/DTG analysis.

ISO Index	Temperature (°C)				
	*t*_5%_	*t*_10%_	*t_max_*_,1_	*t*_90%_	*t_max_*_,2_
138	268.3	294.7	348.7	590.1	600.0
113	264.2	289.4	343.2	539.3	577.1
110	269.1	292.7	347.0	478.0	600.0

**Table 3:** Steady-state coefficient of friction (μ_ss_) values of polyurethane samples under dry and wet sliding conditions.

ISO Index	μ_ss,dry_	μ_ss,wet_	COF Response
138	0.211	0.240	Lowest dry COF
113	0.230	0.176	Lowest wet COF
110	0.238	0.313	Highest wet COF

## Data Availability

The data presented in this study are available on request from the corresponding author.
